# Educated for the region? A register-based sequence analysis modelling medical doctors’ career trajectories

**DOI:** 10.1007/s43999-025-00076-y

**Published:** 2025-10-22

**Authors:** Marie Hella Lindberg, Birgit Abelsen

**Affiliations:** National Centre for Rural Medicine, Department of Community Medicine, UiT Arctic University of Norway, Postbox 6050, Langnes, Tromsø, 9037 Norway

**Keywords:** Career trajectories, Career mobility, Medical workforce, Rural health workforce, Sequence analysis, Cluster analysis

## Abstract

Recruiting and retaining medical doctors in rural and primary healthcare remains a global challenge. Norway and its Northern region are no exception. The medical education programme at UiT The Arctic university of Norway, established to educate for Northern Norway, addressed this challenge early, through initiatives like preferential admission for Northern residents and practice-oriented learning in Northern rural areas. However, previous studies on these interventions have been limited to cross-sectional data, offering little insight into long-term trends. This study leverages longitudinal registry data to map the career trajectories of UiT medical graduates. Using sequence analysis, we modelled doctors’ career paths over a decade by analysing yearly updates on work *states* based on location (Northern or Southern Norway), area type (rural or urban), and healthcare sector (primary or specialist). Cluster analysis identified typical career trajectories, while multinomial logistic regression analysis explored how graduates’ background characteristics were associated with these trajectories. We identified seven distinct career trajectories combining work location, area type and health sector in various ways. Northern-origin doctors largely remained in the North, and those from rural backgrounds were more likely to work in rural areas over time. However, the majority (53.7%) gravitated towards work in urban specialist services, while only 12.8%, dominated by Northern-origin (90%) and rural (83%) backgrounds, pursued careers in Northern rural primary healthcare. These findings underscore the need for targeted educational interventions that account for geographical origins for addressing healthcare personnel recruitment and retention challenges.

**Supplementary Information:**

The online version contains supplementary material available at 10.1007/s43999-025-00076-y.

## Introduction

Understanding the mobility patterns of medical doctors over time is crucial for addressing the global challenge of an uneven geographical distribution of health workers, particularly in rural areas. High turnover rates and persistent shortages have prompted substantial investments in policies aimed at improving rural recruitment and retention. However, gaining a comprehensive understanding of doctors’ mobility remains challenging in the absence of longitudinal, high-quality data [1].

The World Health Organization (WHO) has identified targeted admission policies enrolling students with rural backgrounds in health education programs as a key strategy for supporting rural recruitment [2]. A systematic literature review of medical education interventions identified five primary approaches to recruiting doctors to rural areas, listed from most to least used: practice-oriented learning in rural areas; decentralised education; preferential admission from rural districts; curriculum relevant to rural medicine; and compulsory service periods in rural areas [3]. These interventions are frequently combined, making it difficult to isolate their individual effects. However, the review showed that the proportion of doctors working in a rural area was significantly higher among those covered by interventions compared to those who were not.

The geographical and career mobility of medical doctors is shaped by a complex interplay of factors, including rural background, specialty choice and personal motivations [4–6]. Although rural background is a strong predictor of rural practice, its influence is intertwined with other factors, making it challenging to disentangle [7, 8]. Moreover, many rural doctors do not have rural backgrounds, highlighting the need for broader recruitment strategies [7]. Barriers to the recruitment of urban-origin students include negative perceptions of rural practice and concerns about career progression and isolation [7]. However, rural placements, financial incentives, opportunities for a broader scope of practice and closer doctor-patient relationships can encourage urban-origin students to pursue rural careers [8, 9]. Additional factors, such as familiarity with rural areas, community involvement, and a sense of place, further enhance the intent to practice rurally. Family considerations, including partners’ employment opportunities and educational needs for children, also play a significant role in retention [7, 10–12].

Two opposing forces—*cumulative inertia* and *cumulative stress*—are particularly relevant in understanding doctors’ career trajectories [13–15]. Cumulative inertia refers to the growing attachment to a place over time, driven by social networks and community ties, reducing the inclination to move. Conversely, cumulative stress arises from factors such as job dissatisfaction and limited career opportunities, increasing the propensity to move.

In Norway, the challenges of rural recruitment and retention are especially pronounced in its Northern region. Targeted admission policies have been strategically implemented in the Northern Norwegian educational system for decades. UiT The Arctic University of Norway (UiT) was established in 1972 to expand educational opportunities for Northern Norwegian youth and to recruit professionals, particularly medical doctors, to the region. A 1963 study highlighting the tendency of medical graduates to work where they were raised or educated informed the decision to establish UiT’s undergraduate medical programme [16]. This programme was among the first to offer clinical placements in rural communities, offering placements for practice-oriented learning in both general practice and specialist services throughout Northern Norway. Since its inception, the programme has had an admission quota for applicants from Northern Norway (see Online Resource 1).

Previous research indicates that UiT graduates predominantly work in Northern Norway, both in general practice and specialist services [17–22]. However, these studies are cross-sectional, examining place of work at specific, but arbitrary, points in time. Graduates who may be at different stages in their careers are grouped together, complicating comparison between studies. Cross-sectional designs limit insights into retention, mobility, and career changes. Consequently, there is a need for longitudinal study designs that capture process outcomes such as career *trajectories*, both in terms of geographical location and type of clinical work.

In this paper, we utilise comprehensive longitudinal individual-level data and apply sequence analysis to map and describe the early career trajectories of medical doctors graduated from UiT. This method, first introduced in genetics, was adopted in the social sciences in the 1980s [23]. One of its very first applications was an analysis of musicians’ careers by Abbott and Hrycak [24]. Specifically, we examine doctors’ geographical and career mobility over time and explore the association of key background factors with different career trajectories.

We explore two hypotheses:

### Hypothesis 1

*Doctors with a rural background are more likely to work in a rural area over time*,* either in primary healthcare or specialist health services.*

### Hypothesis 2

*Doctors with Northern backgrounds are more likely to remain in the North over time.*

## Institutional setting

Norway’s national health system is primarily tax-funded, with public healthcare spending per capita among the highest in Europe, accounting for 86% of total health expenditure. The remaining 14% is mainly from out-of-pocket spending, with private health insurance contributing less than 1% [25, 26]. Specialist healthcare services are state-governed and delivered by four regional health authorities, while the 357 municipalities oversee primary healthcare and public health services, including general practitioners (GP), emergency services, nursing homes, and community health centres [23].

Norway has one of the highest numbers of medical doctors per capita globally [23]. Medical doctors are educated at one of four national medical schools or abroad. The primary healthcare system is well-developed, with a high number of GPs per inhabitant, comparable to Scotland and surpassing Denmark, England, and the Netherlands [27]. In 2023, medical doctors’ person-years worked in Norway were approximately 7,200 (26%) in general practice [28, 29], 19,000 (69%) in specialist healthcare services [30] and 1,500 (5%) in other sectors like public administration and research [31].

Most doctors in primary care work within the General Practitioner (GP) scheme, where they are employed by the municipality and assigned a defined resident list. All residents have the right to register with a regular GP, and the majority take advantage of this option. GPs consult with their listed residents, coordinate their care, and act as gatekeepers to secondary care, sick leave, and disability benefits. Regular GPs can either operate as self-employed practitioners—earning income through capitation fees tied to their patient lists and fee-for-service payments—or as salaried employees. Most regular GPs are self-employed, particularly in central, densely populated municipalities. In rural areas, salaried GPs are more common, and there are typically few other doctors besides the regular GPs. Consequently, rural GPs often take on additional municipal responsibilities, such as staffing emergency services, nursing homes, and child health clinics. In more central municipalities, these additional tasks are usually handled by other municipally employed doctors.

To practice clinically for the municipality, doctors must either be specialists in general medicine or in the process of completing their specialisation. In the specialist healthcare service, doctors work in smaller local hospitals or larger regional or university hospitals. Generally, they are salaried employees. They must either be specialists or in the process of specialisation to practice clinically. Larger hospitals typically offer a broader range of medical specialties compared to smaller facilities. Over the past 20 years, the income level of regular GPs has consistently exceeded that of doctors in the specialist healthcare service. Among regular GPs, self-employed practitioners tend to have more listed residents and higher incomes compared to salaried GPs [27].

Public higher education in Norway is free. Medical education lasts six years, followed by specialty training. Internship (mandatory first part of specialty training) includes one year in hospital and six months in general practice. The internship programme provides essential training and aims to ensure an equitable geographical distribution of doctors across the country, supporting recruitment and retention in rural areas [32]. Prior to 2017, it was not mandatory for doctors working in the national health service to be specialists or in specialist training, as it is today.

## Materials and methods

### Data

We utilised data on all graduates from the undergraduate medical programme at UiT from 2003 to 2022 (*N* = 1,705). The study cohort was constructed using UiT’s Common Student Register which linked graduates’ national identity numbers to national registers, including Statistics Norway, The General Practitioner Register, and The Healthcare Personnel Register. This linkage provided comprehensive information on graduates’ socioeconomic and geographical backgrounds, family relationships, and annual updates on employment status, workplace location, and job type post-graduation. Statistics Norway facilitated data linkage by replacing national identity numbers with de-identified IDs for analysis.

The Common Student Register included data on graduates’ gender, age, graduation year, upper secondary school details, and Northern Norwegian quota admission status. Statistics Norway provided information on residence municipality, parents’ education level, marital status, whether graduates had children and workplace data. Work activities were coded using the Standard Industrial Classification (2002 for 2003–2008 and 2007 for 2009–2023). The main employment/contract with the highest employment fraction was used for the analyses. The General Practitioner Register identified regular GPs or locums from 2003 to 2023, while Statistics Norway provided workplace municipality and centrality classification (class 1: most central – class 6: least central) [33]. The Health Personnel Register supplied internship completion dates.

### Ethics

A Data Protection Impact Assessment, conducted with the Norwegian Agency for Shared Services in Education and Research in August 2021, confirmed that data collection, linkage, and storage complied with GDPR regulations (ref.no. 665935).

### Exposures

In line with the study’s hypotheses, the main exposures were doctors’ urban/rural background (centrality class 1–3 or 4–6 at age 16), and doctors’ Northern/Southern Norwegian background (Northern background: residence in the counties Nordland, Troms or Finnmark at age 16, upper secondary school attendance there, or admission via the Northern Norwegian quota).

### Outcome: career trajectories using sequence analysis

To identify career patterns, we conducted sequence analysis. Sequence analysis is considered appropriate for analysing process outcomes like career trajectories [34]. An individual’s career trajectory is defined as a sequence of mutually exclusive categories at each time point, also called “states”. We defined eight states based on the combination of workplace municipality (urban/rural), region (North/South), and type (primary healthcare/specialist services). We added an “Other” state for non-clinical positions and “Missing” for unknown labour market affiliations, resulting in ten mutually exclusive states.

States were updated annually following graduation. We included only cohorts with at least ten years of follow-up after graduation (*N* = 943, graduates from 2003 to 2014), tracking each cohort for ten years. We restricted all individuals’ sequences to an equal length of ten years to study the same early-career period for everyone, and for comparison purposes [35].

To map candidates’ career paths, we applied optimal matching to identify and compare groups of typical career trajectories. Optimal matching is a method used to identify patterns in terms of sequences of states in longitudinal data by calculating the distance, or dissimilarity, between all sequence pairs [24]. The shorter the distance, the more similar the sequences. Optimal matching calculates the distance between sequence pairs as the minimum costs necessary to transform one sequence into another by means of three transformation processes: substitution, insertion and deletion [36]. Substitution occurs when states from one sequence is substituted with states from another to make them equal. Insertion or deletion takes place when states in one sequence are inserted or deleted to make it identical to another. The sum of all transformation processes corresponds to the degree of dissimilarity between sequences [37].

Each transformation is assigned a pre-determined cost. Since one substitution operation can be considered as a combination of one insertion and one deletion, insertion and deletion costs are commonly set to ½ of the maximum substitution cost [36]. In this study, a cost of 1 was assigned to insertions and deletions, and a cost of 2 to substitutions. In this case, the distance between two sequences corresponds to the sum of the minimum number of operations necessary to transform one sequence into another [38].

To identify patterns of similar sequences (career paths), we conducted cluster analysis. We chose the partitioning around medoids approach, as the cluster quality in terms of average silhouette width (see below) was better compared to using hierarchical clustering. A medoid is the observation with the smallest weighted sum of distances from other observations in the group. The partitioning around medoids algorithm minimises the weighted sum of distances from the medoid [39]. To determine the optimal number of clusters, we assessed cluster quality by consulting the average silhouette width. The average silhouette width indicates cluster partitioning quality by comparing an observation’s average weighted distance from the other members of its group and its average weighted distance from the closest other group. Its range is [-1,1], with values close to 1 indicating that the case is more consistent with its own group than with any alternative group of the selected partition [37]. The cluster solution that provides the highest average silhouette width, is the one that has the highest coherence in terms of the strongest within-group homogeneity and the largest between-group differences [39].

We calculated the average time spent in each state by cluster and computed the complexity index as a measure of instability [35] at the cluster level. The index takes values between 0 and 1, where 0 reflects sequences consisting of one single state, and 1 refers to sequences in which all possible states appear in the sequence lasting for an equal amount of time. The complexity of a sequence refers to the degree of instability in how states are arranged within the sequence, reflecting state occurrence, transitions and time spent in each state [40].

#### Robustness checks

Since we had labour market information about the cohorts graduating before 2014 for more than ten years, we reran sequence analysis and cluster analysis allowing for unequal sequence lengths (10–21 years). These were added as supplementary analyses. Moreover, as a robustness check, we repeated the analyses excluding the first two years after graduation, when most internships were completed.

### Statistical analysis: multinomial logistic regression analysis

The clusters resulting from the cluster analysis were used as the dependent variable in two multinomial logistic regression analysis models to study how rural and Northern backgrounds were associated with the likelihood of being assigned to the different clusters (career trajectories). Results were presented as average marginal effects, reflecting the change in likelihood of following a trajectory with a one-unit increase in the predictor variable. This approach was preferred over raw coefficients, as interpreting logits in multinomial logistic regression relative to a reference trajectory is not straightforward. As supplementary analyses, we analysed how marital status and having children at graduation interacted with the exposure variables.

Statistical analyses were conducted using Stata version 17 and RStudio, employing the *TraMineR*, *TraMineRextras*, and *WeightedCluster* packages [39, 41, 42]. A choropleth map was created in Python via Google Colab using *pandas*, *geopandas*, and *matplotlib*. Municipality centrality data was sourced from Statistics Norway, and a GeoJSON file from the Norwegian Mapping Authority was obtained via GitHub [43].

### Covariates

Covariates in the multinomial logistic regression analysis models were age (in years), marital status (married vs. unmarried/divorced/separated), number of children (none vs. one or more) at graduation, and graduation period (early: 2003–2008; late: 2009–2014). These covariates were selected based on statistically significant chi-square tests indicating statistical differences in background variables across clusters (Online Resource 2).

## Results

**Fig. 1:**
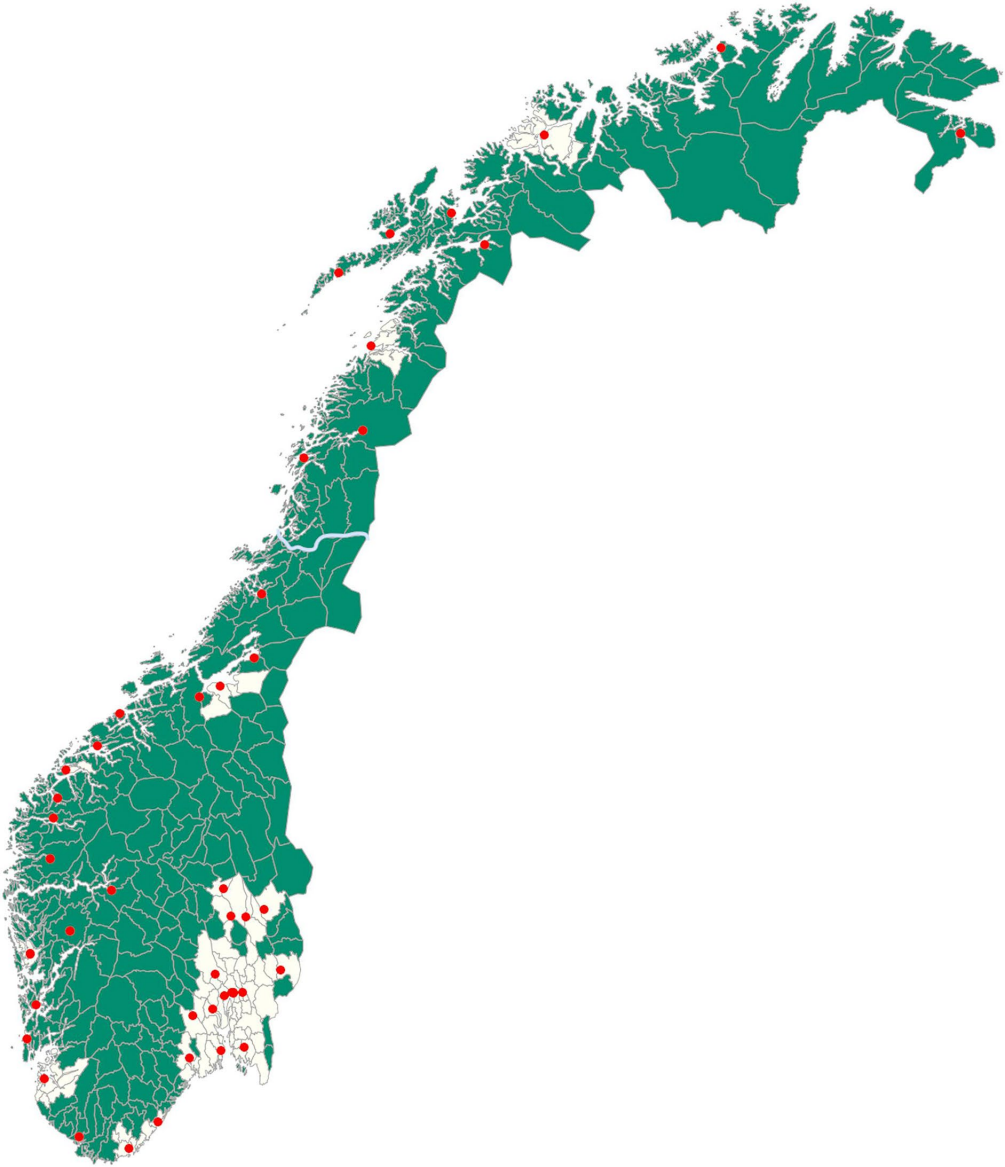
Map of Norway, with urban municipalities colour-coded as light yellow (centrality class 1–3) and rural municipalities colour-coded dark green (centrality class 4–6). The North-South divide is marked by the light blue line. The location of all general hospitals is marked by the red dots

The geographical context of Norway’s healthcare system is illustrated in Fig. 1, showing municipalities color-coded by centrality: dark green for rural and light yellow for urban areas. The North-South divide is marked by the blue line. It is only Bodø and Tromsø that are classified as urban in the North, highlighting its sparse population. The red dots indicate the locations of the 46 general hospitals.

Table 1 reports descriptive characteristics of the 943 medical doctors graduating from UiT between 2003 and 2014. Nearly 75% had a Northern background, 62% had a rural background, and 62% were women.

**Table 1:** Descriptive statistics of the sample

		Male		Female		Total	
		Mean (SD)/%	*N*	Mean (SD)/%	*N*	Mean (SD)/%	*N*
Sex		38.1	359​	61.9​	584​		
Age		28.4 (3.4)	359	28.1 (3.4)	584	28.2 (3.4)	943
Age groups	≤ 27 years	48.2	173	56.3	329	53.2	502
	≥28 years	51.8	186	43.7	255	46.8	441
Marital status at the time of graduation	Married	10.6	38	14.1	82	12.8	120
	Unmarried/divorced/separated	89.4	320	85.9	501	87.2	821
No. of children at the time of graduation	None	77.6	277	69.7	405	72.7	682
	One or more	22.4	80	30.3	176	27.3	256
Parental education	Primary and secondary education ≤ 13 years	23.1	80	30.5	176	27.7	256
	Tertiary education	77.0	267	69.6	402	72.3	669
Rural background	Urban (centrality class 1–3)	39.8	138	36.4	209	37.7	347
	Rural (centrality class 4–6)	60.2	209	63.6	365	62.3	574
Northern background	Yes	73.0	262	73.8	431	73.5	693
	No	27.0	97	26.2	153	26.5	250
Rural and geographical background combined	Rural Northern	51.3	178	54.7	314	53.4	492
	Rural Southern	8.9	31	8.9	51	8.9	82
	Urban Northern	23.9	83	20.0	115	21.5	198
	Urban Southern	15.9	55	16.4	94	16.2	149
Graduation period	2003–2008	50.4	181	46.1	269	47.7	450
	2009–2014	49.6	178	53.9	315	52.3	493
Geographical placement of internship	Entire internship in the north	57.6	181	54.4	275	55.6​	456​
	Partly or entirely in the south	42.4	133	45.7	231	44.4​	364​
Internship in rural municipality	Entirely rural internship	36.3	114	32.0	162	33.7​	276​
	Partly or entirely urban internship	63.7	200	68.0	344	66.43	544​

### Career trajectories

**Fig. 2:**
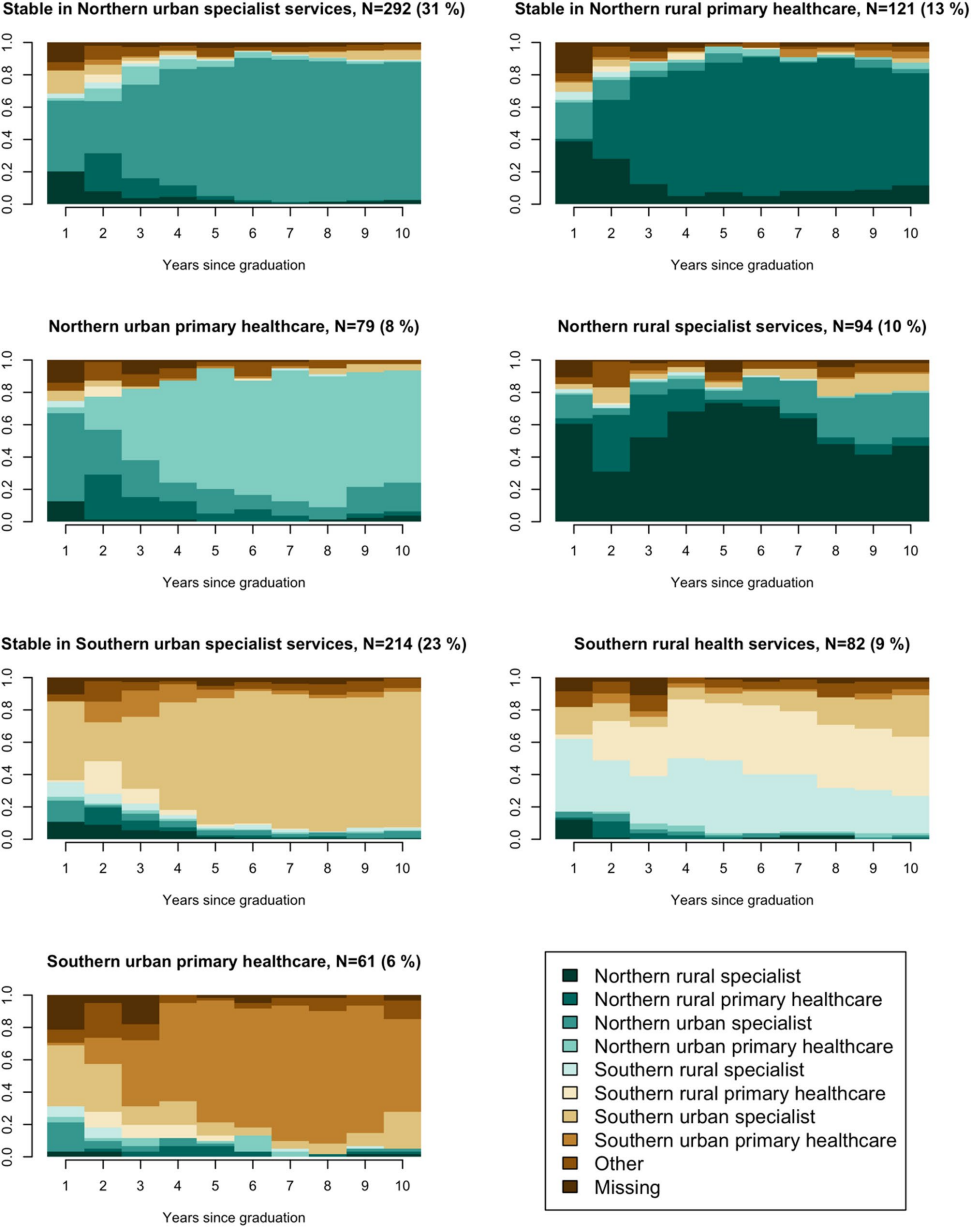
State distribution plot representing the cross-sectional state frequencies for each year

**Fig. 3:**
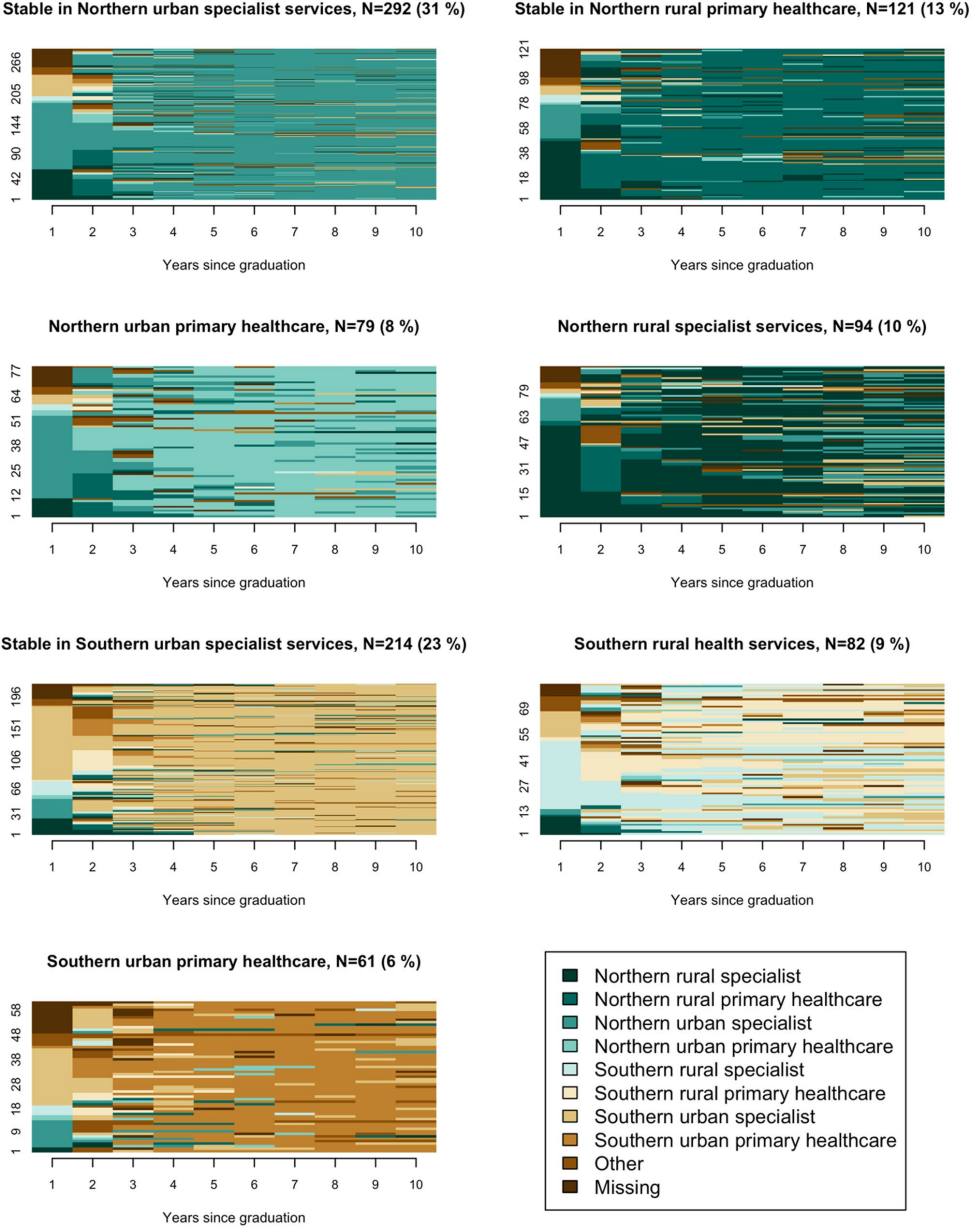
Sequence index plot in which individual sequences are shown as horizontally stacked bars depicting the states over time

Cluster analysis of sequence data identified a 7-cluster solution as the best fit, as this partition achieved the highest average silhouette width of 0.43 compared to other cluster solutions (see Online Resource 3 for plots displaying solutions with 5–8 clusters). These clusters represent seven distinct career trajectories, visualised in Figs. 2 and 3. Figure 2 shows state distribution plots, depicting the cross-sectional proportion of individuals in each state for each year following graduation. Figure 3 presents sequence index plots, in which individual sequences are presented as horizontally stacked bars across the x-axis. It illustrates how individuals transition between regions, urban/rural locations, and job types over the ten-year period. Each individual sequence represents one medical doctor’s career trajectory.

Descriptive characteristics for each cluster are presented in Table 2.

**Table 2:** Descriptive statistics by cluster: means (standard deviation) for continuous and frequencies (proportions) for categorical variables

		Stable in Northern urban specialist services	Stable in Northern rural primary healthcare	Northern urban primary healthcare	Northern rural specialist services	Stable in Southern urban specialist services	Southern rural health services	Southern urban primary healthcare
Silhouette width		0.51	0.47	0.42	0.31	0.46	0.18	0.41
N (%)		292 (31.0%)	121 (12.8%)	79 (8.4%)	94 (10.0%)	214 (22.7%)	82 (8.7%)	61 (6.5%)
Sex	Men	104 (35.6%)	43 (35.5%)	32 (40.5%)	38 (40.4%)	82 (38.3%)	32 (39.0%)	28 (45.9%)
	Women	188 (64.4%)	78 (64.5%)	47 (59.5%)	56 (59.6%)	132 (61.7%)	50 (61.0%)	33 (54.1%)
Age at graduation		28.2 (2.78)	28.7 (4.36)	29.5 (3.714)	27.6 (2.5)	27.4 (3.1)	28.9 (4.4)	28.8 (3.6)
Age groups	≤27	142 (48.6%)	60 (49.6%)	29 (36.7%)	58 (61.7%)	145 (67.8%)	38 (46.3%)	30 (49.2%)
	≥28	150 (51.4%)	61 (50.4%)	50 (63.3%)	36 (38.3%)	69 (32.2%)	44 (53.7%)	31 (50.8%)
Marital status at the time of graduation	Unmarried/divorced/separated	250 (85.6%)	99 (81.8%)	66 (83.5%)	85 (91.4%)	199 (93.4%)	68 (82.9%)	54 (88.5%)
	Married	42 (14.4%)	22 (18.2%)	13 (16.5%)	8 (8.6%)	14 (6.6%)	14 (17.1%)	7 (11.5%)
No. of children at the time of graduation	0	212 (72.6%)	77 (64.2%)	41 (51.9%)	63 (68.5%)	181 (85.0%)	65 (79.3%)	43 (71.7%)
	1 or more	80 (27.4%)	43 (35.8%)	38 (48.1%)	29 (31.5%)	32 (15.0%)	17 (20.7%)	17 (28.3%)
Parental education	Upper secondary	82 (28.6%)	39 (32.5%)	24 (30.8%)	25 (26.9%)	50 (23.9%)	19 (23.5%)	17 (29.8%)
	Tertiary education	205 (71.4%)	81 (67.5%)	54 (69.2%)	68 (73.1%)	159 (76.1%)	62 (76.5%)	40 (70.2%)
Rural background	Urban (centrality class 1–3)	131 (45.6%)	20 (16.7%)	33 (42.3%)	10 (10.8%)	101 (49.0%)	21 (26.2%)	31 (54.4%)
	Rural (centrality class 4–6)	156 (54.4%)	100 (83.3%)	45 (57.7%)	83 (89.2%)	105 (51.0%)	59 (73.8%)	26 (45.6%)
Northern Norwegian background	No	43 (14.7%)	12 (9.9%)	7 (8.9%)	10 (10.6%)	100 (46.7%)	47 (57.3%)	31 (50.8%)
	Yes	249 (85.3%)	109 (90.1%)	72 (91.1%)	84 (89.4%)	114 (53.3%)	35 (42.7%)	30 (49.2%)
Rural and geographical background combined	Rural Northern	147 (51.2%)	97 (80.8%)	43 (55.1%)	79 (84.9%)	78 (37.9%)	25 (31.2%)	23 (40.4%)
	Rural non-Northern	9 (3.1%)	3 (2.5%)	2 (2.6%)	4 (4.3%)	27 (13.1%)	34 (42.5%)	3 (5.3%)
	Urban Northern	102 (35.5%)	12 (10.0%)	29 (37.2%)	5 (5.4%)	34 (16.5%)	9 (11.2%)	7 (12.3%)
	Urban non-Northern	29 (10.1%)	8 (6.7%)	4 (5.1%)	5 (5.4%)	67 (32.5%)	12 (15.0%)	24 (42.1%)
Geographical placement of internship	Partly or entirely in the south	66 (25.8%)	19 (19.4%)	10 (14.7%)	14 (16.7%)	156 (81.7%)	64 (84.2%)	35 (74.5%)
	Entire internship in the north	190 (74.2%)	79 (80.6%)	58 (85.3%)	70 (83.3%)	35 (18.3%)	12 (15.8%)	12 (25.5%)
Internship in rural municipality	Partly or entirely urban internship	199 (77.7%)	48 (49.0%)	55 (80.9%)	25 (29.8%)	148 (77.5%)	29 (38.2%)	40 (85.1%)
	Entirely rural internship	57 (22.3%)	50 (51.0%)	13 (19.1%)	59 (70.2%)	43 (22.5%)	47 (61.8%)	7 (14.9%)
Graduation period	2003–2008	121 (41.4%)	51 (42.1%)	53 (67.1%)	45 (47.9%)	108 (50.5%)	37 (45.1%)	35 (57.4%)
	2009–2014	171 (58.6%)	70 (57.9%)	26 (32.9%)	49 (52.1%)	106 (49.5%)	45 (54.9%)	26 (42.6%)

Table 3 shows the average time spent in each state, the average number of transitions between states and the complexity by career trajectory. Additionally, Online Resource 4 reports the mean complexity of each cluster according to Northern and rural backgrounds.

**Table 3:** Mean time spent in each state and average number of transitions by cluster (career trajectory)

	Cluster/career trajectory						
*​State*	Stable in Northern urban specialist health services	Stable in Northern rural primary healthcare	Northern urban primary healthcare	Northern rural specialist health services	Stable in Southern urban specialist health services	Southern rural health services	Southern urban primary healthcare
Northern Norway rural specialist	0.46	1.34	0.24	5.56	0.35	0.20	0.10
Northern Norway rural primary healthcare​	0.49	6.54	0.75	1.04	0.24	0.20	0.26
Northern Norway urban specialist	7.18	0.60	1.91	1.55	0.41	0.22	0.36
Northern Norway urban primary healthcare	0.41	0.28	5.80	0.09	0.10	0.16	0.25
Southern Norway rural specialist	0.1​4	0.09	0.06	0.11	0.32	3.41	0.16
Southern Norway rural primary healthcare	0.08	0.09	0.08	0.01	0.37	3.24	0.30
Southern Norway urban specialist	0.43	0.14	0.24	0.66	6.74	1.29	1.49
Southern Norway urban primary healthcare​	0.07	0.20	0.05	0.06	0.57	0.26	5.71
Other​	0.43	0.33	0.54	0.59	0.54	0.67	0.80
Missing​	0.3​3	0.40	0.33	0.33	0.36	0.35	0.57
Average no. of transitions	2.97	3.24	3.70	3.98	3.32	3.62	3.89
Complexity	0.324	0.360	0.413	0.430	0.357	0.408	0.431

Common for all clusters was an initial phase of frequent transitions between states, reflecting the completion of internships at the beginning of doctors’ careers. The high proportion of individuals in specialist service states during the first year corresponded to the mandatory 12 months of hospital training, while the relatively large share of doctors in primary healthcare states in the second year reflected the six-month general practice training (Figs. 2 and 3).

Three clusters demonstrated relative stability, with individuals spending an average of 6.5–7.2 years in a single dominant state. These clusters were characterised by slightly fewer transitions and lower complexity scores (< 0.4), indicating a tendency to stabilise over time, with shorter periods spent in other states in some trajectories. In contrast, the remaining clusters exhibited more frequent transitions between states, as indicated by a higher number of transitions, greater complexity, and more frequent spells in states other than the cluster’s most prevalent one.

Clusters were named based on trajectory patterns: (1) Stable in Northern urban specialist service, (2) Stable in Northern rural primary healthcare, (3) Northern urban primary healthcare, (4) Northern rural specialist service, (5) Stable in Southern urban specialist service, (6) Southern rural health services, and (7) Southern urban primary healthcare.

**Fig. 4:**
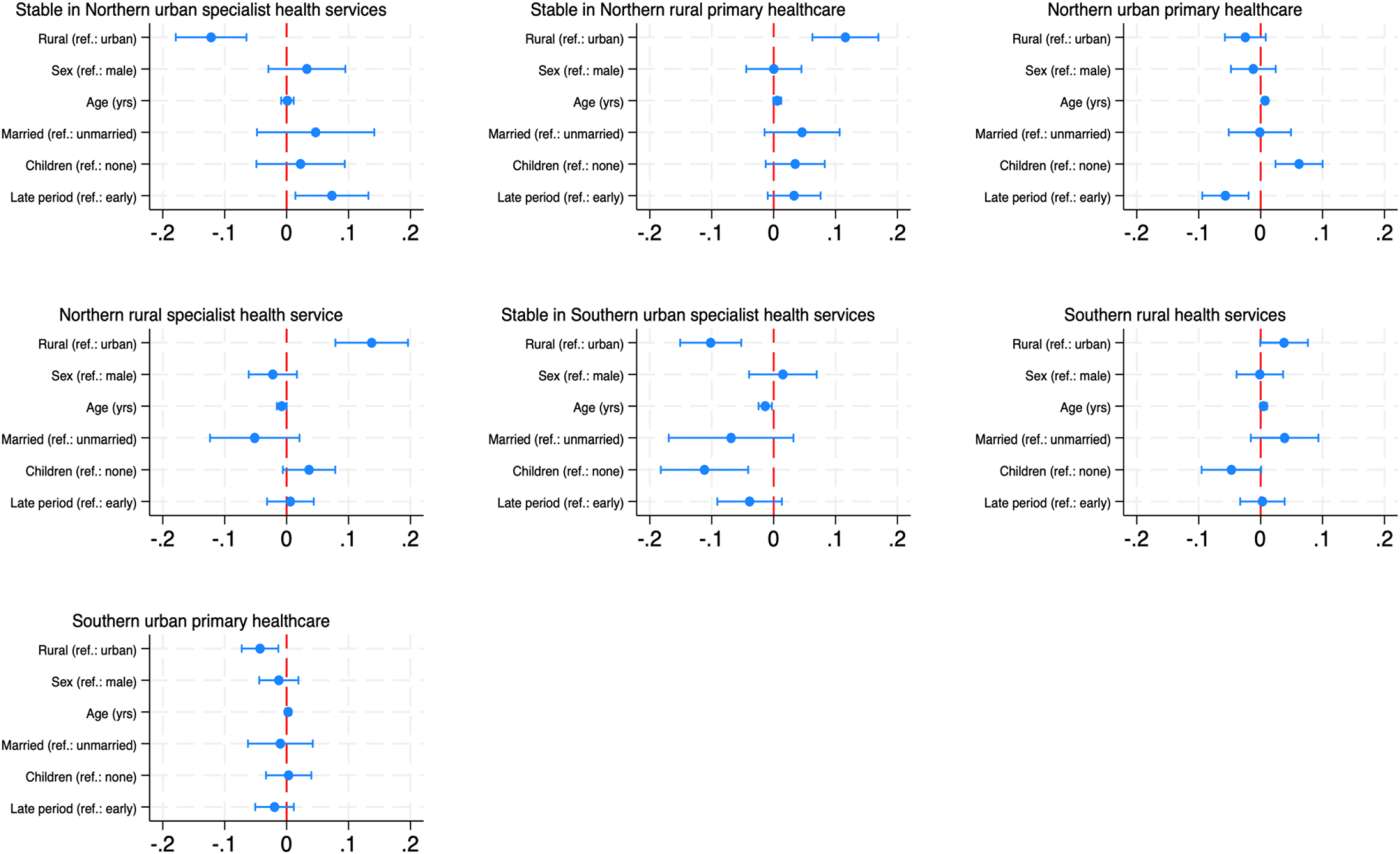
Average marginal effects of rural background on cluster assignment. Average marginal effects show the change in probability when the predictor increases by one unit. For continuous variables (e.g., age) this represents the average change for each additional unit. For binary variables (e.g., urban/rural), the change is from 0 to 1

**Fig. 5:**
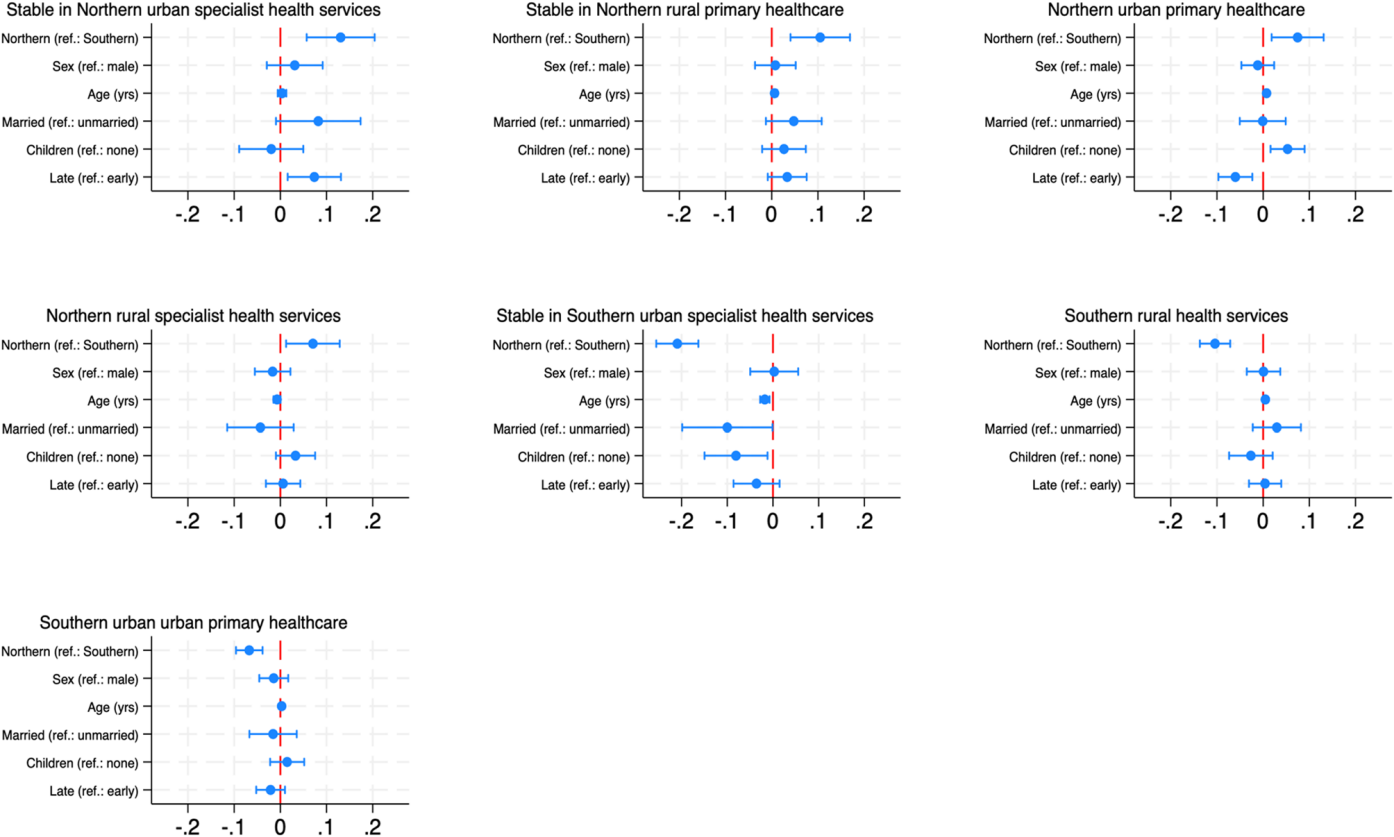
Average marginal effects of Northern Norwegian background on cluster assignment. Average marginal effects show the change in probability when the predictor increases by one unit. For binary variables (e.g., Northern/Southern background), the change is from 0 to 1

Multinomial logistic regression models, reported as average marginal effects (AME), assessed how having rural or Northern backgrounds were associated with the likelihood of being assigned to the different clusters, controlling for sex, age at graduation, study period, having children, and marital status (Figs. 4 and 5). The results indicated that a rural background increased the likelihood of assignment to predominantly rural clusters, relative to an urban background. Similarly, a Northern background, relative to a Southern background, was positively associated with the likelihood of being in the Northern clusters, while the association was negative for the Southern clusters. The raw coefficients from the multinomial logistic regression analyses are presented in Online Resources 5 and 6. The cluster-specific associations are further described below.

#### Stable in Northern urban specialist services

This cluster, the largest in the sample (31%), included individuals primarily working in specialist healthcare in Northern urban areas, spending an average of 7.2 years in this state. It exhibited the lowest average number of transitions (2.97) and the lowest complexity (0.32), suggesting that individuals tended to stabilise in the dominant state over time, particularly after the first two years, during which most doctors complete their internships.

Candidates largely completed internships in Northern (74%) or urban (78%) municipalities, and the majority had Northern backgrounds (85%). This cluster had the smallest proportion of individuals with rural backgrounds (54%) among the Northern clusters (Table 2). Doctors with rural backgrounds had a 12% lower likelihood than urban-origin doctors (AME: -0.12, 95% CI [-0.18, -0.06]) to follow this trajectory, while those with Northern backgrounds were 13% more likely than those with Southern backgrounds to do so (AME: 0.13, 95% CI [0.06, 0.20]). This was the only cluster where the associations of rural and Northern backgrounds were in opposite directions. This may reflect the relatively high proportion of doctors with urban backgrounds completing internships in urban areas.

#### Stable in Northern rural primary healthcare

This cluster was characterised by careers in primary healthcare in Northern rural areas, averaging 6.5 years in this state. The relatively few transitions and low complexity suggest a comparably stable career trajectory in which a considerable portion seemed to stabilise in rural primary healthcare in a Northern rural municipality after year one, in which nearly 70% of individuals were registered working in specialist healthcare, which was likely reflected by the completion of the hospital training component of their internships. It had the highest proportion of married graduates (18%). Having Northern (90%) and rural (83%) backgrounds dominated. Rural vs. urban and Northern vs. Southern backgrounds increased the likelihood of following this trajectory by 12% (AME: 0.12, 95% CI [0.06, 0.17]) and 11%, respectively (AME: 0.11, 95% CI [0.04, 0.17]). Internships were predominantly completed in the North (81%).

#### Northern urban primary health care

This cluster, representing 8.4% of the sample, involved work in primary healthcare in Northern urban areas, with an average of 5.8 years spent in this state. The transition rate (3.70) and complexity (0.41) were higher, which can reflect the considerable amount of time spent in the Northern Norway urban specialist trajectory (nearly two years, particularly during the first two years, but also towards the end of the study period). It included the highest share of graduates with children (48%) and those aged 28 or older (63%). Having a Northern background dominated at 91%, with a 7% higher likelihood of following this trajectory compared to Southern-origin doctors (AME: 0.07, 95% CI [0.02, 0.13]). There were 58% with a rural background, which was not significantly associated with assignment to this cluster (AME: -0.02, 95% CI [-0.06, 0.01]). Internships were mainly completed in the North (85%) in urban municipalities (81%).

#### Northern rural specialist services

Individuals spent an average of 5.6 years working in the specialist services in Northern rural municipalities in this cluster, comprising 10% of the sample. There was also a considerable occurrence of other North-based states, e.g., Northern urban specialist services (1.6 years), especially towards the end of the study period. There were frequent transitions between states at an average rate of 3.98, and among the highest complexity across clusters (0.43). The trajectory was dominated by Northern (89%) and rural (89%) backgrounds. Rural backgrounds increased the likelihood of this trajectory by 14% relative to urban backgrounds (AME: 0.14, 95% CI [0.08, 0.20]), and Northern backgrounds by 7% relative to Southern backgrounds (AME: 0.07, 95% CI [0.01, 0.13]). Most internships were completed in the North (83%) and in rural municipalities (70%).

#### Stable in Southern urban specialist services

This cluster included 23% of the sample, with individuals working in Southern urban specialist services for an average of 6.7 years. Career trajectories seemed to stabilise in this state over time after more frequent transitions the first few years. This cluster also displayed stability in terms of relatively low transitions (3.32) and complexity (0.36). It had the lowest share of graduates with children (15%). Rural backgrounds decreased the likelihood of assignment to this trajectory by 10% compared to urban backgrounds (AME: -0.10, 95% CI [-0.15, -0.05]), and Northern backgrounds by 21% relative to Southern backgrounds (AME: -0.21, 95% CI [-0.26, -0.16]). Most doctors completed their internships in the South (82%) and urban areas (78%).

#### Southern rural health services

This cluster was dominated by work in Southern rural municipalities with an average of 3.4 and 3.2 years spent in primary healthcare and specialist service jobs, respectively. A substantial share of approximately 80% worked in specialist healthcare the first year, followed by a considerable share transitioning to primary healthcare. There were few sequences of only one state, although the average individual transition rate was only slightly higher than the more stable clusters at 3.62. Complexity was relatively high (0.41). A rural background was prevalent (74%) but was not significantly associated with assignment to this trajectory (AME: 0.04, 95% CI [0.00, 0.08]). Southern-origin doctors constituted 57% of the cluster, and having a Northern background was associated with a 10% lower likelihood of following this trajectory (AME: -0.10, 95% CI [-0.14, -0.07]). Most internships were completed in the South (84%), and 62% in rural municipalities. This cluster had the lowest silhouette width (0.18).

#### Southern urban primary healthcare

The smallest cluster involved work in Southern urban primary healthcare, averaging 5.7 years in this state. This cluster displayed instability, particularly the first years, as few individuals started the career trajectories in the dominant state. There was a relatively high transition rate (3.89) and the highest complexity (0.43). The composition of urban and Southern backgrounds was 54% and 61%, respectively. Rural backgrounds decreased the likelihood of following this trajectory by 4% compared to urban ones (AME: -0.04, 95% CI [-0.07, -0.01]), and Northern backgrounds by 7% compared to Southern backgrounds (AME: -0.07, 95% CI [-0.10, -0.04]). The majority completed their internship in the South (75%) and in urban municipalities (85%).

#### Robustness checks

Excluding the first two years post-graduation resulted in only minor changes in cluster assignment, indicating that the first years of a doctor’s career are formative for subsequent stages (Online Resource 7). The average silhouette width suggested that nine clusters would be optimal (0.51 vs. 0.53 for seven vs. nine clusters, respectively).

Including the full sequence lengths for all included cohorts (10–21 years) resulted in a similar overall picture with the same 7-cluster partitioning (average silhouette width = 0.44) as in the analyses with equal sequence length. The main difference was a more apparent overall stability as the duration and prevalence of the dominant state was greater (see state distribution and sequence index plots in Online Resources 8 and 9).

Analysing whether marital status and having children at graduation interacted with rural and Northern backgrounds identified some statistically significant interactions, particularly for the assignments to the Southern trajectories. E.g., interacting rural background with having children had an AME of -0.11 (95% CI [-0.18, -0.05]) (Online Resource 10).

## Discussion

This study has shed light on the career trajectories of medical doctors from UiT, revealing key trends in early career choices and geographical mobility. Rather than focusing solely on where doctors “end up”, the use of sequence analysis allowed us to capture how typical career trajectories unfold, accounting for variations in order, timing and duration. This longitudinal approach offered a more comprehensive understanding of how medical graduates’ careers evolve over time compared to studies examining career mobility at isolated time points [16–22]. Our findings revealed that a substantial proportion of graduates remained in Northern Norway, with the majority originating from the region. There was an inclination toward urban specialist services, including those with rural backgrounds—a trend that may pose challenges for rural recruitment and retention efforts. Nevertheless, our findings supported both hypotheses 1 and 2: a rural or Northern background was positively associated with pursuing careers in rural municipalities or the North, respectively. These findings underscore the importance of regional attachment for career choices, lending support to the introduction of preferential admission to applicants from e.g., rural areas.

One clear advantage of sequence analysis is its ability to identify patterns in the timing, duration, and order of states. In our study, we observed distinct patterns, such as states reflecting the completion of internships early in the career and clusters with frequent transitions between states, potentially indicating challenges in securing permanent positions. However, unlike other studies applying sequence analysis to study career trajectories, there is no inherent order to the states in our context—none of the states logically precede others. For example, Vinkenburg et al. [44] studied research career trajectories, where the order of states reflected upward mobility within the hierarchy of research positions. In contrast, the states in our study do not follow a hierarchical or sequential structure.

Career trajectories varied in terms of stability, with some paths characterised by frequent transitions and others by relative steadiness. Trajectories with high variability and complexity often involved transitions between states within the same region (North or South) and with similar centrality (urban or rural) as the most prevalent state. This suggests that instability was primarily driven by shifts between specialist and primary healthcare jobs, rather than geographical mobility. While transitions within the same region may be less disruptive, those requiring relocation—such as for specialist training in larger hospitals—are likely to impose greater cumulative stress [14], negatively affecting retention.

Among the identified career trajectories, instability was particularly evident in the Northern urban primary healthcare and Northern rural specialist services trajectories. This may reflect challenges in securing long-term contracts or meeting specialist training requirements. Similarly, in the Southern rural health services path, rural specialist and rural primary healthcare states were equally prevalent. This pattern may indicate frequent job changes within the same geographical area, potentially driven by job dissatisfaction [45] or competition for permanent positions.

Graduates, including those from rural backgrounds, tended to favour specialist service jobs in urban settings. While this could reflect the greater availability of specialist positions in urban areas, there may be discrepancies between the supply of medical doctors and the demand for healthcare services in rural areas. This may be driven by a focus on “hospital specialties”, while the demand for primary healthcare skills is the most apparent in rural areas [46, 47]. However, the presence of many smaller hospitals in rural municipalities underscores a demand for specialist skills also in rural areas.

Factors influencing these career choices include lifestyle preferences, attractiveness of rural practice, exposure to different medical disciplines, professional opportunities and support in rural areas. While financial incentives are common tools to boost rural recruitment and retention, non-monetary factors like professional support, family and community engagement have been found to be equally important [7, 48, 49]. This suggests that focusing solely on rural-origin doctors may be too narrow [7, 50]. Nonetheless, our finding that rural backgrounds were positively associated with working in rural areas supports the introduction of admission quotas for rural applicants [3], particularly in Norway, where most municipalities are rural.

Our findings highlight the need for strategies to enhance rural practice attractiveness and address the motivations influencing career choices. The positive impact of preferential admission for Northern-origin applicants on regional doctor supply, as well as the mandated creation and distribution of internship positions [32], suggests that targeted strategies are essential for addressing healthcare workforce challenges. International studies indicate that inadequate childcare and educational opportunities for children are significant factors driving doctors to leave rural positions [11, 12]. While similar evidence from Norway is lacking, it is reasonable to assume that these factors also influence Norwegian doctors’ decisions to remain in rural areas.

Norway’s well-developed childcare system, with capped day-care fees for all and free day-care in Northern regions (Finnmark and Northern Troms), aims to attract and retain professionals. When it comes to primary schools, quality indicators suggest that certain characteristics—such as a central location, a high proportion of parents with higher education, a significant share of teachers meeting formal qualification standards, and teacher stability—positively impact school quality [51]. These characteristics are often less common in rural areas. Secondary schools’ centralised structure often forces rural youth to relocate for further schooling. Addressing these non-professional needs, such as enhancing educational opportunities for children and youth, could be a viable policy to improve rural doctor retention.

Additionally, the Framework for Remote Rural Workforce Stability [52] outlines nine strategic elements across three tasks: plan, recruit, and retain. Priorities include implementing appropriate service models, providing relocation support for recruits and their families, fostering team cohesion, and offering tailored training for rural careers.

In recent years, all four Norwegian universities offering medical education have decentralised their programmes, establishing campuses in more rural areas across the country. Early evidence suggests that doctors who choose a decentralised track are often from the local area and tend to remain there after completing their education [22]. It remains to be seen whether this trend will have a lasting impact and whether it applies to all decentralised study tracks.

### Strengths and limitations

This study offers a novel application of sequence analysis to examine medical doctors’ career trajectories —a method that, to our knowledge, has not previously been applied to this profession. Utilising rich, linked register data from multiple sources, we provide insights into mobility trends that can inform recruitment and retention policies. Moreover, the study contributes to rural health workforce research within a European context, a field dominated by research from Australia, the US, and Canada [50].

This study has several limitations that should be acknowledged. First, our study population is subject to selection bias due to UiT’s quota system, favouring students with Northern, often rural, backgrounds. This limits the generalisability of our findings. Second, the lack of detailed data on rural *exposure* during internships limits our ability to explore factors that might explain why some urban-origin doctors choose to work in rural areas. Third, the study did not account for temporal variability in doctor demand and supply, which likely affects career and mobility choices. Fourth, municipalities defined as rural (centrality classes 4–6) differ substantially: class 4 municipalities have midsize towns with access to a range of services, while class 6 municipalities are sparsely populated with long distances to urban centres and services. Grouping these municipalities may obscure the vastly different work realities. Fifth, the identified career trajectories provide a broad representation of relatively similar individual trajectories, but some individual trajectories may not align well with the overall characterisation. Sixth, the 7-cluster partitioning resulted in an average silhouette width of 0.43, which would indicate a weak data structure, as strong structures typically have average silhouette widths of ≥0.75 [39]. However, analyses grouping sequences based on relatively many variables tend to achieve only moderate silhouette widths [37]. Seventh, the observed instability in career trajectories suggests a need for qualitative methods to examine *why* medical doctors transition between states, which is an important next step for further exploration.

## Conclusions

This study identifies key trends in early career trajectories among UiT medical graduates, confirming our hypotheses that rural or Northern backgrounds are associated with a greater likelihood of working in rural areas or the North. While many graduates remain in Northern Norway, a significant number work in urban specialist services. It remains uncertain whether this trend comes at the expense of rural communities’ healthcare needs, warranting further investigation using appropriate methods. Our findings indicate that UiT’s preferential admission for Northern-origin applicants has been successful, highlighting the importance of targeted strategies that consider geographical origins influencing career decisions. Sequence analysis effectively captures the dynamic nature of career trajectories, providing insights crucial for developing a stable and equitable distribution of medical doctors across both urban and rural areas.

## Supplementary Information

Below is the link to the electronic supplementary material.

Supplementary Material 1

## Data Availability

The data that support the findings of this study contain sensitive information. Therefore, restrictions apply to the availability of these data, which were used under license for the current study and are not publicly available. According to Norwegian law and the General Data Protection Regulation, the authors are not permitted to share the datasets used in this study with third parties.
