# Supplementary material for: Educated for the region? A register-based sequence analysis modelling medical doctors’ career trajectories
Source: Res Health Serv Reg. 2025 Oct 22;4:16. doi: 10.1007/s43999-025-00076-y (PMC12545940; doi:10.1007/s43999-025-00076-y)
Supplement: Supplementary file 1 — Supplementary Material 1 [file 43999_2025_76_MOESM1_ESM.pdf]

Online Resource 1

The Northern Norwegian admission quota

At UiT The Arctic University of Norway, certain study programmes have admission quotas for applicants from Northern Norway, which includes the counties of Nordland, Troms, and Finnmark. This initiative is part of a strategy to enhance access to higher education for young people in Northern Norway and to ensure a skilled workforce in the region. The underlying hypothesis is that students are more likely to remain in the area if they can pursue their studies where they grew up.

The medical education programme is one such programme with a preferential admission quota for applicants from Northern Norway. This quota has been in place since the first cohort of medical students was admitted in 1973. Initially, 25% of the spots were reserved for applicants with a Northern affiliation. In 1998, this increased to 60%. To qualify for this admission quota, applicants must demonstrate their connection to the region by meeting at least three of the following criteria:

- Certification of residence in Northern Norway
- Completion of upper secondary school in one of the three Northern counties
- A lower secondary diploma from a Northern municipality
- Residency in the region for at least ten years
- At least one parent currently living or having lived in the region within the past ten years
- The mother's place of residence was in the region at the time of the applicant's birth

Online Resource 2

Chi-square tests of statistical significance between career trajectories according to categories of background variables

		N	Share	*p* value chi-sqr
**Sex**	Men	359	0.38	0.794
	Women	584	0.62	
**Age at the time of graduation**	27 years and younger	502	0.53	<0.001
	28 years and above	441	0.47	
**Marital status at the time of graduation**	Unmarried/divorced/separated	821	0.87	0.020
	Married	120	0.13	
**No. of children at the time of graduation**	No children	682	0.73	<0.001
	One or more	256	0.27	
**Parental education**	Upper secondary	256	0.28	0.645
	Tertiary	669	0.72	
**Northern Norwegian background**	Yes	693	0.74	<0.001
	No	250	0.26	
**Centrality background**	Urban (cl 1-3)	347	0.38	<0.001
	Rural (cl 4-6)	574	0.62	
**Graduation period**	2003-2008	450	0.48	0.002
	2009-2014	493	0.52	
**Internship in the north**	Enitrely/partly in the south	364	0.44	<0.001
	Entirely in the north	456	0.56	
**Internship in a rural municipality**	Enitrely/partly in an urban municipality	544	0.66	<0.001
	Entirely in rural municipality	276	0.34	

Online Resource 3

Five clusters

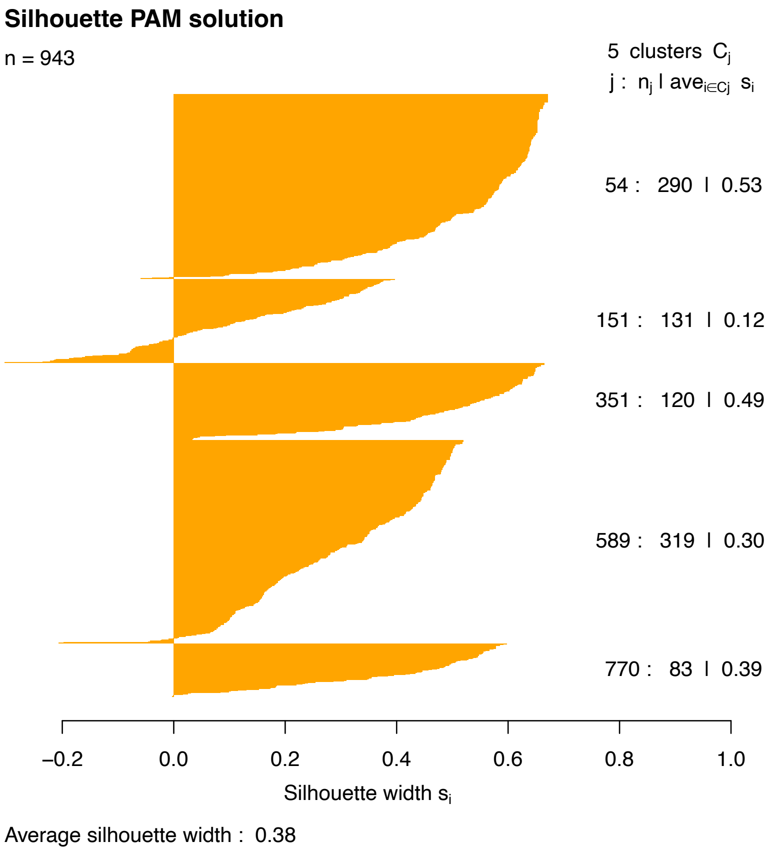

Six clusters

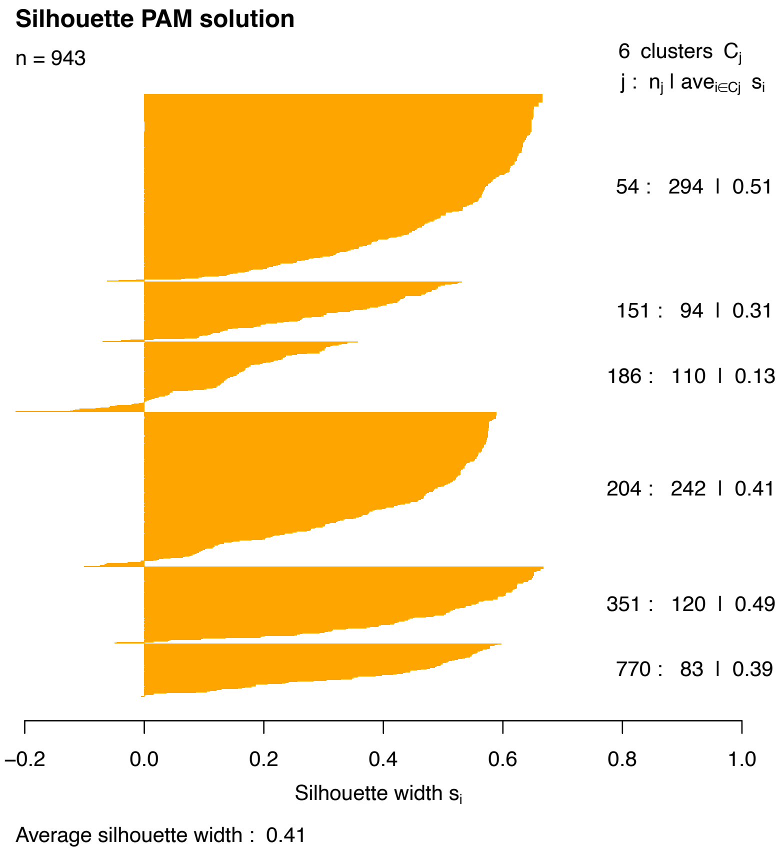

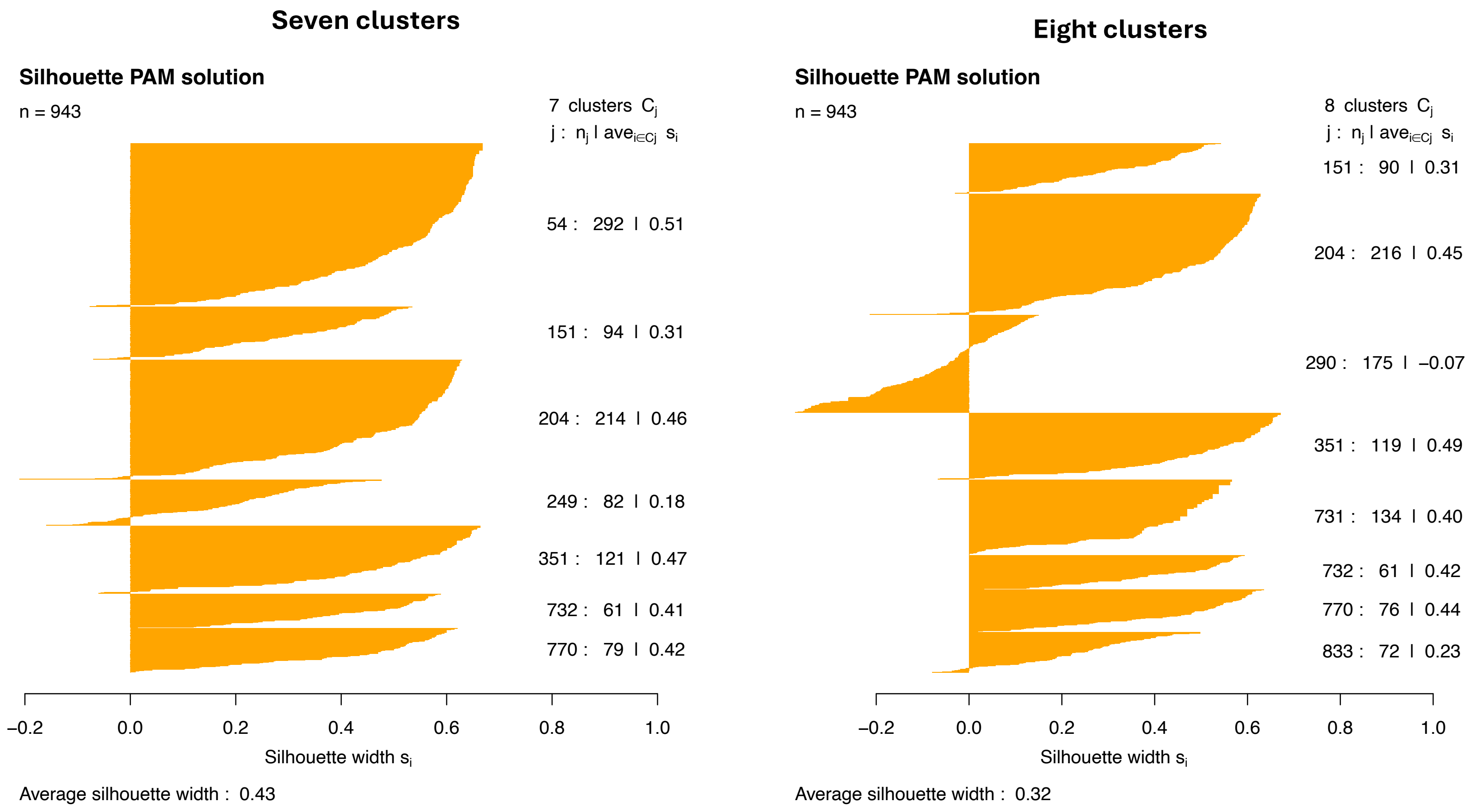

Plots of four different cluster solutions, reporting how well sequences are grouped in the clusters.

Online Resource 4

Complexity[a] by cluster in total and according to Northern and rural backgrounds

	Total	Northern background		Rural background	
		Yes	No	Yes	No
**Stable in Northern urban specialist services**	0.324	0.322	0.337	0.326	0.320
**Stable in Northern rural primary healthcare**	0.360	0.349	0.462	0.358	0.361
**Northern urban primary health care**	0.413	0.398	0.570	0.433	0.382
**Northern rural specialist services**	0.430	0.431	0.417	0.432	0.406
**Stable in Southern urban specialist services**	0.357	0.364	0.350	0.361	0.352
**Southern rural health services**	0.408	0.420	0.399	0.402	0.451
**Southern urban primary healthcare**	0.431	0.454	0.409	0.439	0.437

[a] The index takes values between 0 and 1, where 0 reflects sequences consisting of one single state, and 1 refers to sequences in which all possible states appear in the sequence lasting for an equal amount of time. The complexity of a sequence refers to the degree of instability in how states are arranged within the sequence [b]

[b] Ritschard G. Measuring the Nature of Individual Sequences. Sociological Methods & Research. 2023;52(4):2016-49. https://doi.org/10.1177/00491241211036156).

Online Resource 5

Multinomial logistic regression model for the relative probability of belonging to the clusters as a function of **rural background**[a,b]

	**Stable in Northern urban specialist services** vs stable in Southern urban specialist services	**Stable in Northern rural primary healthcare** vs stable in Southern urban specialist services	**Northern urban primary healthcare** vs stable in Southern urban specialist services	**Northern rural specialist services** vs stable in Southern urban specialist services	**Southern rural health services** vs stable in Southern urban specialist services	**Southern urban primary healthcare** vs stable in Southern urban specialist services
**Rural background** (ref.: urban background)	**0.096**<br>0.186	**1.518*****<br>0.287	**0.214**<br>0.278	**2.017*****<br>0.364	**0.998*****<br>0.293	**-0.250**<br>0.303
Age (years)	0.068*<br>0.037	0.113***<br>0.042	0.155***<br>0.043	-0.016<br>0.055	0.120***<br>0.046	0.104**<br>0.051
Sex (ref.: men)	0.035<br>0.194	-0.080<br>0.250	-0.222<br>0.283	-0.308<br>0.267	-0.091<br>0.278	-0.274<br>0.308
Married (ref.: unmarried/ divorced)	0.475<br>0.340	0.699*<br>0.393	0.319<br>0.440	-0.195<br>0.508	0.780*<br>0.442	0.155<br>0.535
One or more children (ref.: none)	0.600**<br>0.248	0.839***<br>0.297	1.317***<br>0.320	0.922***<br>0.317	-0.004<br>0.367	0.580<br>0.380
Graduation period: 2009-2014 (ref.: 2003-08)	0.415**<br>0.187	0.452*<br>0.242	-0.522*<br>0.286	0.257<br>0.261	0.223<br>0.269	-0.156<br>0.304
Intercept	-2.435**<br>1.086	-5.674***<br>1.286	-5.014***<br>1.303	-2.246<br>1.613	-5.289***<br>1.400	-3.794**<br>1.519
No. of observations	*918*					

[a]Reference cluster: stable in Southern urban specialist services.
[b]All models are adjusted for age, sex, marital status at the time of graduation, number of children at the time of graduation, and whether candidates graduated early/late in the observation period.
*** p<0.01, ** p<0.05, * p<0.1

Online Resource 6

Multinomial logistic regression model for the relative probability of belonging to the clusters as a function of **Northern background**[a,b]

	**Stable in Northern urban specialist services** vs stable in Southern urban specialist services	**Stable in Northern rural primary healthcare** vs stable in Southern urban specialist services	**Northern urban primary healthcare** vs stable in Southern urban specialist services	**Northern rural specialist services** vs stable in Southern urban specialist services	**Southern rural health services** vs stable in Southern urban specialist services	**Southern urban primary healthcare** vs stable in Southern urban specialist services
**Northern background** (ref.: urban background)	**1.692***** (0.222)	**2.170***** (0.345)	**2.269***** (0.435)	**2.023***** (0.381)	**-0.320** (0.269)	**-0.113** (0.301)
Age (years)	0.102*** (0.037)	0.142*** (0.041)	0.187*** (0.043)	0.016 (0.054)	0.139*** (0.043)	0.130*** (0.047)
Sex (ref.: men)	0.095 (0.198)	0.056 (0.251)	-0.154 (0.287)	-0.178 (0.265)	-0.004 (0.273)	-0.241 (0.300)
Married (ref.: unmarried/ divorced)	0.787** (0.354)	0.916** (0.405)	0.527 (0.457)	0.072 (0.513)	0.800 (0.434)	0.213 (0.521)
One or more children (ref.: none)	0.388 (0.253)	0.684** (0.298)	1.140*** (0.323)	0.797** (0.317)	0.049 (0.367)	0.611 (0.373)
Graduation period: 2009-2014 (ref.: 2003-08)	0.436** (0.192)	0.462* (0.244)	-0.549 (0.292)	0.256 (0.260)	0.202 (0.266)	-0.184 (0.299)
Intercept	-4.605*** (1.120)	-7.178*** (1.293)	-7.523*** (1.383)	-3.269** (1.616)	-5.107*** (1.307)	-4.576*** (1.445)
No. of observations	*938*					

[a]Reference cluster: stable in Southern urban specialist services.
[b]All models are adjusted for age, sex, marital status at the time of graduation, number of children at the time of graduation, and whether candidates graduated early/late in the observation period.
*** p<0.01, ** p<0.05, * p<0.1

Online Resource 7

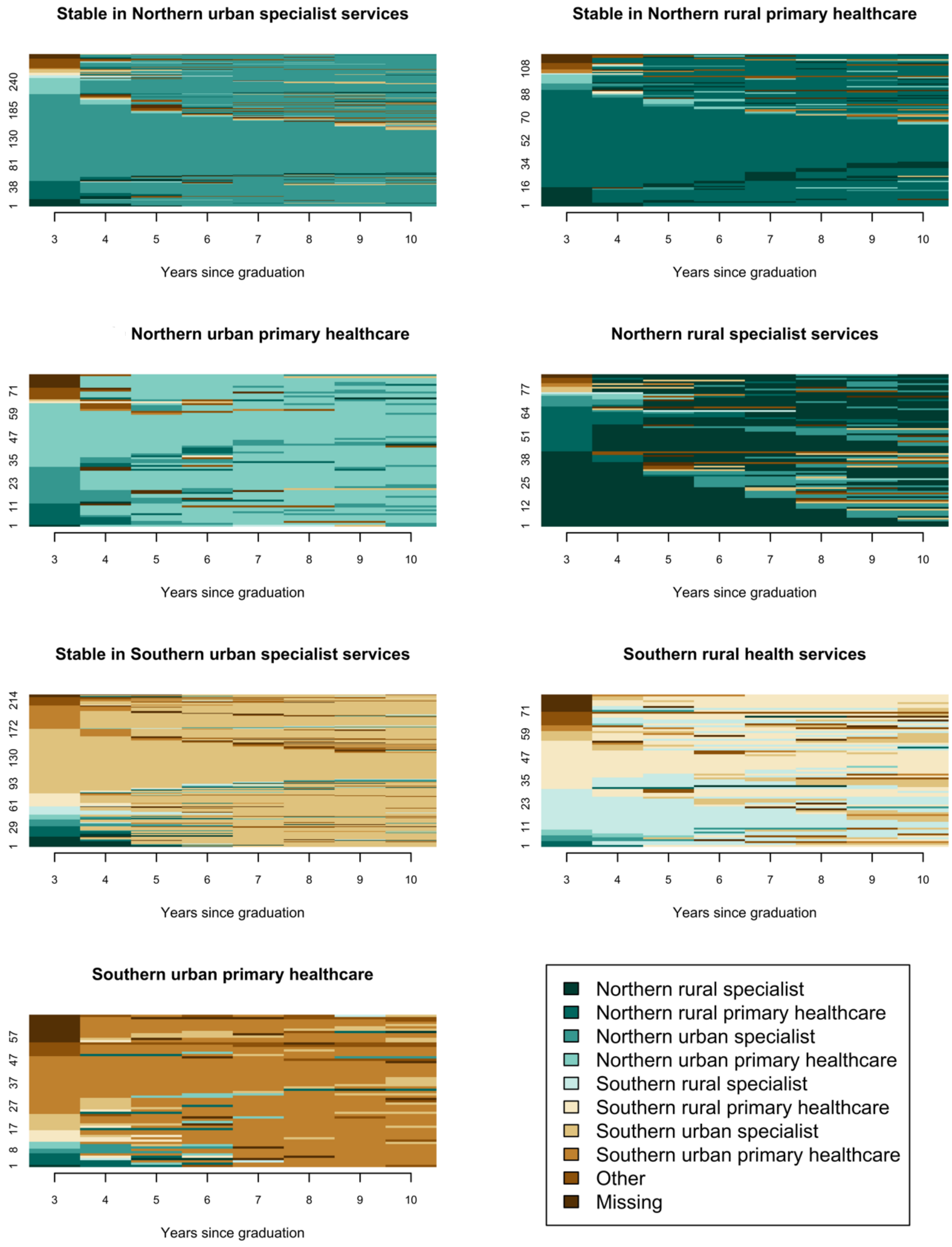

Sequence index plot of clusters removing the first two years

Online Resource 8

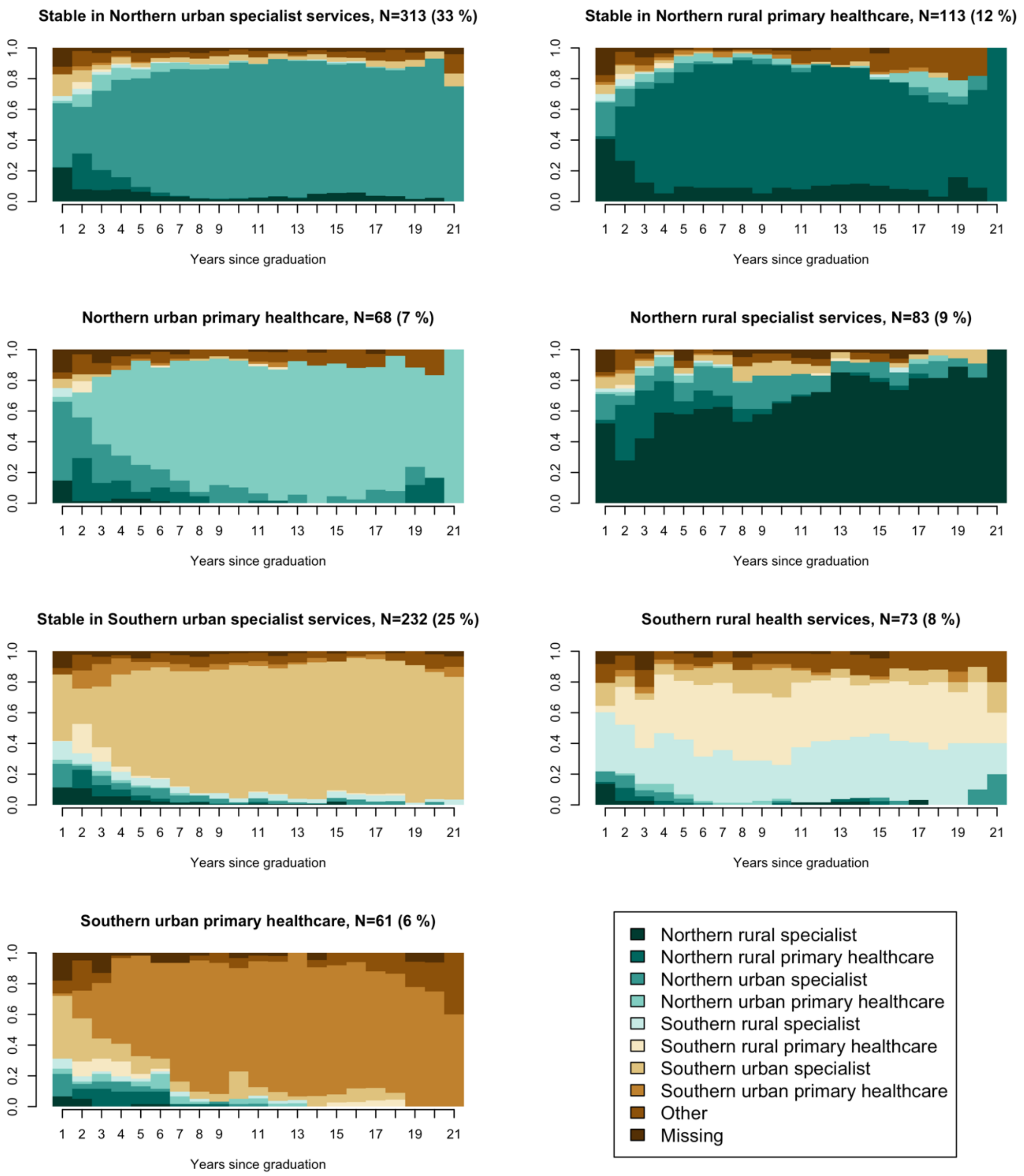

Online Resource 9

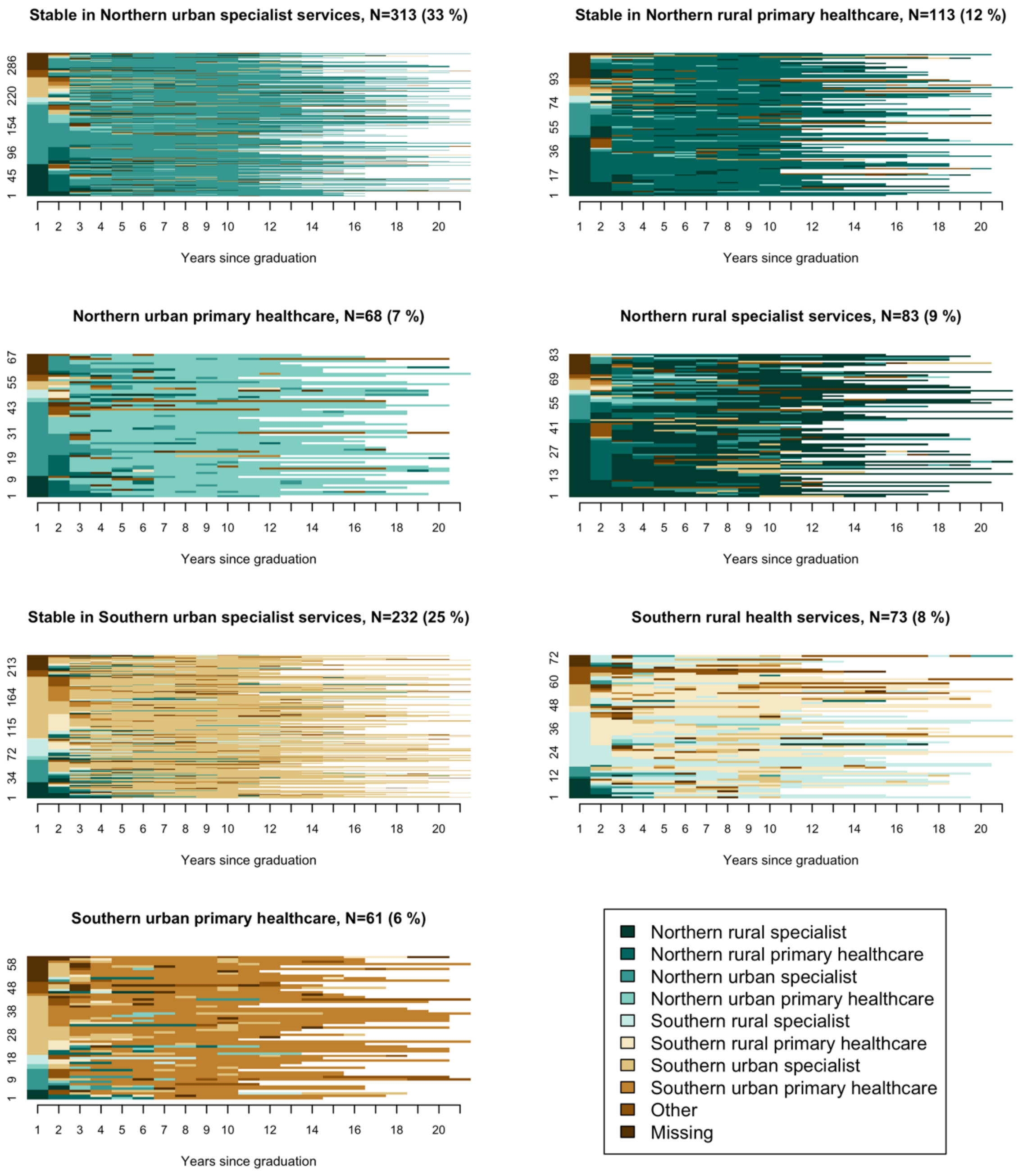

Online resource 10

Average marginal effects of rural and Northern Norwegian backgrounds interacted with marital status and having children at graduation on cluster assignment.

	**Stable in Northern urban specialist services**	**Stable in Northern rural primary healthcare**	**Northern urban primary healthcare**	**Northern rural specialist services**	**Stable in Southern urban specialist services**	**Southern rural health services**	**Southern urban primary healthcare**
Rural background given being married	0.078<br>(0.063)<br>[-0.045, 0.201]	0.073<br>(0.054)<br>[-0.034, 0.180]	-0.009<br>(0.029)<br>[-0.066, 0.049]	**-0.085**<br>(0.034)<br>[-0.152, -0.017]	-0.082<br>(0.047)<br>[-0.173, 0.010]	0.060<br>(0.052)<br>[-0.043, 0.162]	**-0.036**<br>(0.017)<br>[-0.070, -0.002]
Rural background given having one or more children	-0.013<br>(0.042)<br>[-0.096, 0.069]	0.059<br>(0.038)<br>[-0.016, 0.134]	0.061<br>(0.028)<br>[0.006, 0.115]	0.054<br>(0.037)<br>[-0.018, 0.127]	**-0.112**<br>(0.032)<br>[-0.175, -0.050]	**-0.059**<br>(0.026)<br>[-0.110, -0.009]	0.011<br>(0.021)<br>[-0.029, 0.052]
Northern Norwegian background given being married	0.085<br>(0.062)<br>[-0.037, 0.206]	0.062<br>(0.049)<br>[-0.034, 0.159]	0.001<br>(0.033)<br>[-0.063, 0.065]	-0.048<br>(0.034)<br>[-0.114, 0.018]	-0.070<br>(0.042)<br>[-0.153, 0.012]	0.006<br>(0.030)<br>[-0.052, 0.064]	**-0.036**<br>(0.015)<br>[-0.065, -0.007]
Northern Norwegian background given having one or more children	-0.058<br>(0.041)<br>[-0.138, 0.022]	0.039<br>(0.033)<br>[-0.026, 0.103]	**0.069**<br>(0.028)<br>[0.014, 0.124]	0.037<br>(0.030)<br>[-0.022, 0.097]	**-0.066**<br>(0.029)<br>[-0.123, -0.008]	**-0.035**<br>(0.016)<br>[-0.067, -0.003]	0.014<br>(0.019)<br>[-0.023, 0.050]

Note: Average marginal effect estimates statistically significant at the 95% level in **bold**. Standard errors in parentheses and 95% confidence intervals in square brackets.
